# Complete Genomic Analysis of a *Salmonella enterica* Serovar Typhimurium Isolate Cultured From Ready-to-Eat Pork in China Carrying One Large Plasmid Containing *mcr-1*

**DOI:** 10.3389/fmicb.2018.00616

**Published:** 2018-04-27

**Authors:** Wei Wang, Zulqarnain Baloch, Mingyuan Zou, Yinping Dong, Zixin Peng, Yujie Hu, Jin Xu, Nafeesa Yasmeen, Fengqin Li, Séamus Fanning

**Affiliations:** ^1^Key Laboratory of Food Safety Risk Assessment, Ministry of Health, China National Center for Food Safety Risk Assessment, Beijing, China; ^2^College of Veterinary Medicine, South China Agricultural University, Guangzhou, China; ^3^Heilongjiang Provincial Center for Disease Control and Prevention, Harbin, China; ^4^Institute of Microbiology, University of Agriculture Faisalabad, Faisalabad, Pakistan; ^5^UCD-Centre for Food Safety, School of Public Health, Physiotherapy and Sports Science, University College Dublin, Dublin, Ireland; ^6^Institute for Global Food Security, School of Biological Sciences, Queen’s University Belfast, Belfast, Ireland

**Keywords:** MDR *Salmonella enteric* serovar Typhimurium, conjugation, mcr-1, *phoP/Q*, *pmrA/B*, plasmids, ready-to-eat pork

## Abstract

One *mcr-1*-carrying ST34-type *Salmonella* Typhimurium WW012 was cultured from 3,200 ready-to-eat (RTE) pork samples in 2014 in China. Broth dilution method was applied to obtain the antimicrobial susceptibility of *Salmonella* Typhimurium WW012. Broth matting assays were carried out to detect transferability of this phenotype and whole-genome sequencing was performed to analyze its genomic characteristic. Thirty out of 3,200 RTE samples were positive for *Salmonella* and the three most frequent serotypes were identified as *S*. Derby (*n* = 8), *S*. Typhimurium (*n* = 6), and *S*. Enteritidis (*n* = 6). One *S*. Typhimurium isolate (*S*. Typhimurium WW012) cultured from RTE prepared pork was found to contain the *mcr-1* gene. *S*. Typhimurium WW012 expressed a level of high resistance to seven different antimicrobial compounds in addition to colistin (MIC = 8 mg/L). A single plasmid, pWW012 (151,609-bp) was identified and found to be of an IncHI2/HI2A type that encoded a *mcr-1* gene along with six additional antimicrobial resistance genes. Plasmid pWW012 contained an IS*30*-*mcr-1*-*orf*-*orf*-IS*30* composite transposon that can be successfully transferred to *Escherichia coli* J53. When assessed further, the latter demonstrated considerable similarity to three plasmids pHYEC7-*mcr-1*, pSCC4, and pHNSHP45-2, respectively. Furthermore, plasmid pWW012 also contained a multidrug resistance (MDR) genetic structure IS*26*-*aadA2*-*cmlA2*-*aadA1*-IS*406*-*sul3*-IS*26*-*dfrA12*-*aadA2*-IS*26*, which showed high similarity to two plasmids, pHNLDF400 and pHNSHP45-2, respectively. Moreover, genes mapping to the chromosome (4,991,167-bp) were found to carry 28 mutations, related to two component regulatory systems (*pmrAB, phoPQ*) leading to modifications of lipid A component of the lipopolysaccharide structure. Additionally, one mutation (D87N) in the quinolone resistance determining region (QRDR) gene of *gyrA* was identified in this *mcr-1* harboring *S*. Typhimurium. In addition, various virulence factors and heavy metal resistance-encoding genes were also identified on the genome of *S*. Typhimurium WW012. This is the first report of the complete nucleotide sequence of *mcr-1*-carrying MDR *S*. Typhimurium strain from RTE pork in China.

## Introduction

*Salmonella* is often acquired from contaminated food, and is an important zoonotic bacterium linked to cases of gastroenteritis and bacteremia. This bacterium is now the leading cause of global bacterial food poisoning out breaks and is associated with increased morbidity and mortality (Kirk et al., 2015). There are estimated to be 94 million cases of gastroenteritis globally per year, with 155,000 deaths attributed to this bacterium (Majowicz et al., 2010; Gallati et al., 2013). It was reported that between 1994 and 2005, approximately 22% of food borne infections in China were caused by *Salmonella* (Wang et al., 2007). Of note, *Salmonella* Typhimurium is one of the most prevalent serovars identified among these food borne illnesses in China (Zhang et al., 2014). *Salmonella* Typhimurium strains are most commonly isolated from retail meat particularly pork in China, and both sporadic and outbreak-associated cases of human salmonellosis caused by this bacterium often need clinical therapy (Zhang et al., 2016). Moreover, this bacterium has the potential to act as a reservoir for different antimicrobial resistance-encoding genes (Zhang et al., 2016).

The relevance of *S.* Typhimurium is also marked by its capability to acquire resistance determinants, to various drug classes, especially those that are third-generation cephalosporins, tetracyclines, (fluoro) quinolones, folate pathway inhibitors, phenicols, penicillines, aminoglycosides and macrolides (Wong et al., 2014; Yi et al., 2017). Resistance to polymyxin in bacteria including *Salmonella* is commonly due to chromosomal mutations in two component regulatory systems (e.g., *pmrAB* and *phoPQ*) resulting in structural modification of lipid A. Therefore, bacterial resistant to polymyxin was historically thought to be rare (Lee et al., 2014). However, recently, a new plasmid-encoding polymyxin resistance gene, *mcr-1*, was discovered from bacterial isolated from the environment, animals, and humans in China (Liu et al., 2016). Subsequently, occurrence of the *mcr-1* gene has been reported worldwide, indicating a newly recognized global distribution. Furthermore, the *mcr-1*-bearing plasmids were found to possess the ability to disseminate between food-producing animal and human bacteria and were also highly stable in these backgrounds, even in the absence of polymyxin selection (Anjum et al., 2016; Kempf et al., 2016; Liu et al., 2016; El Garch et al., 2017).

The *mcr-1* gene in *Salmonella* was originally detected from human and food in England and Wales, including 8 *S*. Typhimurium, 1 *S.* Paratyphi B var Java and 1 *S*. Virchow strains (Doumith et al., 2016). Since then, this gene was reported from *Salmonella* isolates in Europe, the United States, and China, from humans, food-producing animals, and their surrounding environment, and retail meat (Anjum et al., 2016; Campos et al., 2016; Figueiredo et al., 2016; Quesada et al., 2016; Vinueza-Burgos et al., 2016; Yang et al., 2016). Nonetheless, to date, few reports of the detection of *mcr-1* have been cited in *Salmonella* from ready-to-eat (RTE) pork meat in China. In this study, we report for the first time, the prevalence of *Salmonella* species among RTE pork in China, and while one *S*. Typhimurium isolate was then found to be positive for the *mcr-1* gene, which was located on a large transferable plasmid, along with multiple antimicrobial resistance genes. The complete genome sequence of both the plasmid and chromosome was determined to shed insight into the diversity and complexity of this interesting strain.

## Materials and Methods

### Strains Isolation

**FIGURE 1:**
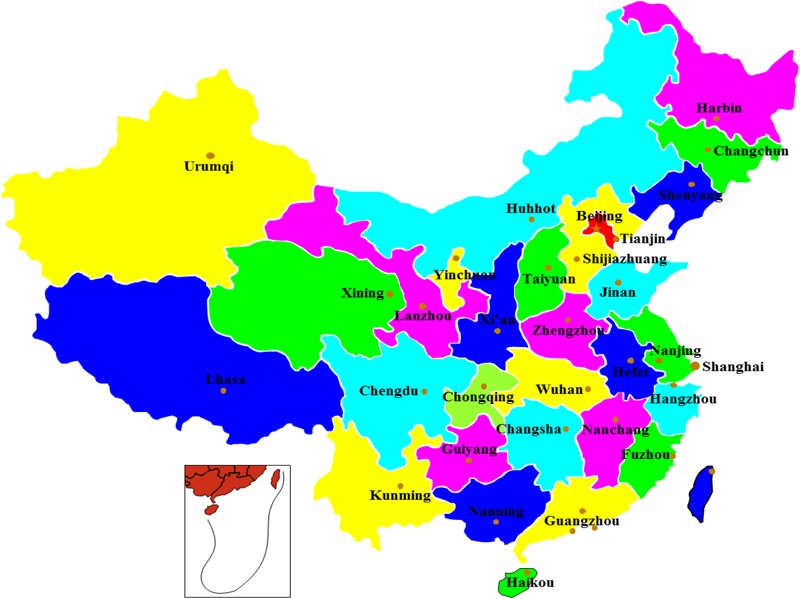
Map of China showing the locations of the 32 metropolitan cities from where the *Salmonella* isolates were collected.

A total of 3,200 RTE pork samples were collected from 32 retail outlets and 32 commercial hypermarkets, in 32 provincial capitals of China in 2014 (**Figure 1**). Fifty RTE pork samples were collected at each sampling site and all were stored inside tightly sealed aseptic bags, surrounded by a biological ice bag, and then placed in a box maintained at a temperature lower than 4°C. Samples were immediately transported to the laboratory and subjected to microbiological analysis within 2 h. All samples were subjected to qualitative analysis for *Salmonella* using an enrichment method described by the National Food Safety Standard of China-Food microbiological examination, *Salmonella* (GB 4789.4-2010). Finally, presumptive *Salmonella* were selected for biochemical confirmation using API 20E test identification test strips (bioMérieux, Marcy l′ Etoile, France), as well as for molecular identification using PCR assay targeting the *invA* gene (Malorny et al., 2003). For all of the confirmed *Salmonella* isolates, serotypes were determined by the slide agglutination test, using *Salmonella* antisera (Statens Serum Institute, Denmark) according to the Kauffmann–White scheme. All confirmed *Salmonella* isolates were stored in brain heart infusion broth with 40% [v/v] glycerol (Land Bridge, Beijing, China) at -80°C. Each sample retained was represented by at least one bacterial isolate.

### Detection of *mcr-1* Gene

The frozen strains were cultured overnight at 37°C in brain heart infusion broth. A TIANamp Bacterial DNA extraction kit (TianGen DNA Kit DP302, Beijing, China) was used to extract the genomic DNA from the culture according to the manufacturer’s instructions. A NanoDrop-2000 spectrophotometer (Thermo Fisher Scientific, NH, United States) was then used to evaluate the quality of DNA. PCR was performed to confirm the presence of the *mcr-1* gene among the studied strains using primers targeting *mcr-1* as reported previously (Liu et al., 2016). The genetic identity of all amplicons was subsequently confirmed by nucleotide sequencing (Liu et al., 2016). Finally, one *S*. Typhimurium isolate designated as *S*. Typhimurium WW012 was determined to harbor the *mcr-1* gene and this isolate was selected to perform the follow-up experiments.

### Antimicrobial Susceptibility Tests (AST)

Antimicrobial susceptibility testing (AST) of the *S*. Typhimurium WW012 isolate was performed using broth dilution method, using the Biofosun^®^ Gram-negative panel (Fosun Diagnostics, Shanghai, China). Data obtained was interpreted according to standards and guidelines described by Clinical and Laboratory Standards Institute [CLSI] (2016). Additionally, we also used the National Antimicrobial Resistance Monitoring System (NARMS) protocol when CLSI standards were not available. The panel of 21 antimicrobial compounds as shown in **Table 1** was tested Reference *Escherichia coli* strain ATCC^TM^25922 was used as the quality control.

**Table 1:** Antimicrobial resistance test of *S.* Typhimurium WW012 to a panel of 21 antimicrobial agents^∗^.

Class	Antimicrobial agent^a^	MIC (mg/L)	R/I/S
Penicillins	Ampicillin (AMP)	1	S
Cephalosporins	Cefepime (FEP)	0.125	S
	Cefoxitin (CFX)	2	S
	Cefazolin (CFZ)	1	S
	Cefotaxime (CTX)	0.125	S
	Ceftazidime (CAZ)	0.25	S
	Ceftiofur (TIO)^b^,€	0.25	S
Carbapenems	Imipenem (IMI)	0.5	S
	Meropenem (MEM)	0.125	S
Aminoglicosides	Gentamicin (GEN)	1	S
	Streptomycin (STR)^b^	> 32	R
Polymyxin	Colistin (CT)€	8	R
beta-lactamase inhibitor combinations	Amoxicillin/Clavulanic Acid (AMC)	>64/32	R
Folate pathway inhibitors	Trimethoprim/sulfamethoxazole (SXT)	>8/152	R
Tetracyclines	Tetracycline (TET)	>32	R
Phenicols	Chloramphenicol (CHL)	32	R
	Florfenicol (FFC)€	>16	R
Fluoroquinolones	Nalidixic acid(NAL)	>128	R
	Ciprofloxacin (CIP)	0.12	I
	Enrofloxacin (ENO)€	0.125	S
Macrolides	Azithromycin (AMZ)€	4	S

*^∗^Data was interpreted according to (a) CLSI; (b) NARMS; €Used as a feed additive in animal production.*

### Conjugal Plasmid Transfer and Characterization

The *mcr-1* harboring *S*. Typhimurium WW012 isolate was analyzed for its ability to transfer the colistin resistance phenotype using broth matting to the plasmid-free recipients, *E. coli* J53. Transfer of *mcr-1* to transconjugants was confirmed by PCR (Cui et al., 2017). Plasmid DNA profiles of both the donor and transconjugants were carried out using the S1-nuclease digestion pulsed-field gel electrophoresis (S1-PFGE) molecular sub-typing method (Liu et al., 2016).

### Whole Genome Sequencing and Annotation

To assess the genomic background of *S*. Typhimurium WW012, whole-genome sequencing (WGS) was performed using the SMRT^®^ Pacific Biosciences RS II platform (Pacific Biosciences, Menlo Park, CA, United States). Annotation of the genomes was performed using RAST^1^, BLASTn and BLASTp^2^ programs. The open reading frame (ORF) Finder program^3^ was used to identify features of public health interest. PlasmidFinder-1.3^4^ was used to identify plasmid replicon types. The comprehensive antibiotic resistance database (CARD)^5^ was used to identify antimicrobial resistance genes. Virulence factor database (VFDB)^6^ was queried to predict the presence of virulence factors.

Three complete genomes, *Salmonella* Typhimurium LT2 (Accession number AE006468.1), *Salmonella* Typhi CT18 (AL513382.1), and *Salmonella* Indiana C629 (Accession number CP015724) were available from GenBank and used to compare the *Salmonella* pathogenicity islands (SPIs) with *S*. Typhimurium WW012 in this study by SPIFinder^7^. Finally, the antibacterial biocide and metal resistance genes database (BacMet)^8^ was used to predict the presence of any metal-resistance genes.

### Nucleotide Accession Numbers

The complete genome of *S*. Typhimurium WW012 was deposited at GenBank under the Accession number CP022168 (chromosome) and CP022169 (plasmid p WW012).

## Results

### Prevalence of *mcr-1*-Positive *Salmonella*

In this study, we collected and tested a total of 3,200 RTE pork samples from 32 provincial capitals in China in 2014. Thirty (30/3,200, 0.94%) samples were positive for *Salmonella*; one isolate from each sample was selected for subsequent analyses. In total, thirty isolates were recovered (comprising 10 serotypes), and the three most frequent serotypes among RTE pork samples covering 66.7% of the total, were identified as *S*. Derby (*n* = 8), *S*. Typhimurium (*n* = 6), and *S*. Enteritidis (*n* = 6). One (1/30, 3.3%) *S*. Typhimurium isolate (designated as *S*. Typhimurium WW012) was cultured from RTE prepared pork collected from a convenience market in Nanning city, Guangxi Province, and found to contain the *mcr-1* gene. The sampling details of isolates are shown in **Supplementary Table S1**.

### Susceptibility to Antimicrobial Compounds

In this study, *S*. Typhimurium WW012 expressed a level of high resistance to seven different antimicrobial compounds in addition to colistin (MIC = 8 mg/L) (**Table 1**). The isolate was susceptible to all tested penicillins, cephalosporins, carbapenems, and macrolides (**Table 1**). Moreover, *S*. Typhimurium WW012 was also found to be susceptible to enrofloxacin, whilst expressing an intermediate resistant phenotype to ciprofloxacin (**Table 1**).

### Horizontal Transfer of *mcr-1* Genes and Associated Determinants

One plasmid was identified from *S.* Typhimurium WW012 by S1-PFGE and the conjugation assay showed that it could be transferred to a plasmid-free recipient *E. coli* J53 with a conjugation frequency of 1.2 × 10^-6^ as determined under laboratory conditions (**Table 2** and **Supplementary Figure S1**). MIC value of the transconjugant were recorded at 8mg/L using broth dilution test, which showed 64-fold increase in when compared with the recipient *E. coli* J53 (0.125 mg/L). This transconjugant expressed a MDR phenotype and was found to be resistance to five antimicrobial compounds including of trimethoprim, sulfamethoxazole, streptomycin, tetracycline, chloramphenicol, and florfenicol, in addition to colistin.

**Table 2:** The summary of the features associated with the genome and plasmid identified in *S.* Typhimurium WW012^∗^.

Location	Size (bp)	G+C%	ORFs	RNAs	ST/Inc type	S1-PFGE	Conjugation frequency	Antimicrobial resistance phenotype	Antimicrobial resistance genes
Chromosome	4,991,167	52.1%	4,684	110	ST34	-	-	Streptomycin	*strA,strB*
								Sulfamethoxazole	*sul2*
								Tetracycline	*tet(B)*
								Nalidixic acid	*gyrA* (D87N)
								Colistin	PhoPQ *phoP* (S131P), *phoQ* (T168A); *ArnBCADTEF*operson *arnA* (L48Q, G51E, N184D, A195S, I247M, G284D, C303R, D337N, E554D, D631G), *arnC*(S320P), *arnD* (P164S, V216A); PmerCAB *pmrC* (I81V, Q415E), *pmrA* (S89T, R211G), *pmrB* (T18M, S76G, V86I, T114A); *pmrD* (L85S)
Plasmid	151,609	45.0%	168	-	*IncHI2, IncHI2A*	∼150 kb	1.2 × 10^-6^	Streptomycin	*aadA1, aadA2*
								Sulfamethoxazole	*sul3*
								Trimethoprim	*dfrA12*
								Colistin	*mcr-1*
								Chloramphenicol, florfenicol	*cmlA1*

*^∗^No features were identified.*

### Genome Sequence Information

The *S*. Typhimurium WW012 genome consisted of a single circular chromosome and a circular plasmid (**Table 2** and **Supplementary Figure S2**). The chromosome consisted of 4,991,167 bp with 4,684 predicted ORFs along with 110 RNAs. Meanwhile, a single circular extra-chromosomal element was identified and denoted as plasmid pWW012 of IncHI2 and IncHI2A replicon types. The total size of plasmid pWW012 was 151,609 bp including 168 predicted ORFs with the average GC content of 45.0% (**Table 2**). Moreover, the multilocus sequence type (MLST) of *S*. Typhimurium WW012 was identified as ST34 (**Table 2**).

### Acquired Antimicrobial Resistance-Encoding Genes Identified in the Bacterial Genome

The CARD database was queried to identify resistance related genotypes on the genome of *S*. Typhimurium WW012. A total of 21 antimicrobial resistance genes (two genes were identified as *aadA2*) were identified, which encoded resistance to 11 antimicrobial agents of 10 different classes, including 14 genes on the chromosome and 7 on the plasmid (**Table 2** and **Supplementary Figure S2**). In detail, when compared with *S*. Typhimurium LT2, one missense mutation in *gyrA* (D87N) and a total of 24 missense mutations in the PmrAB and PhoPQ systems were found within the chromosome of *S*. Typhimurium WW012. Moreover, four and five additional antimicrobial resistance genes of *strA/B, sul2, tet(B)* were also found within the chromosome and plasmid, respectively, (**Table 2** and **Supplementary Figure S2**).

### Virulence Factors Annotated in the Bacterial Genome

To identify potential virulence-encoding genes in *S*. Typhimurium WW012, the genome was used to query those factors listed in the VFDB. These were aligned to the ORF protein sequences using BLASTP and filtered with 90% identity and match length. Using this approach, 201 virulence factors were identified on the chromosome, among which 66 genes belonged to type-three secretion system (T3SS) encoding genes (**Supplementary Figure S2** and **Supplementary Table S2**). The genomes of *S.* Typhimurium WW012 were also compared with those of *S*. Typhimurium LT2, *S*. Typhi CT18, and *S*. Indiana C629 in NCBI to identify the presence of SPIs. It was found that *S.* Typhimurium WW012 possessed SPI-3, SPI-4, SPI-5, SPI-13, SPI-14, and C63PI (**Table 3**) in addition to SPI-1 and SPI-2. Moreover, 63 fimbrial adherence-encoding genes and other virulence-encoding genes were identified on the chromosome of *S.* Typhimurium WW012 (**Supplementary Figure S2** and **Supplementary Table S2**).

**Table 3:** Distribution of SPIs in four representative genomes of *Salmonella* stains^∗^.

Genomic Island	*S*. Typhimurium WW012	*S.* Typhimurium LT2	*S.* Typhi CT18	*S.* Indiana C629
SPI-1	+	+	+	+
SPI-2	+	+	+	+
SPI-3	+	+	+	-
SPI-4	+	+	+	+
SPI-5	+	+	+	+
SPI-6	-	-	+	-
SPI-7	-	-	+	-
SPI-8	-	-	+	-
SPI-9	-	-	+	-
SPI-10	-	-	+	-
SPI-12	-	-	+	-
SPI-13	+	+	-	-
SPI-14	+	+	-	-
C63PI	+	+	-	+

*^∗^Not identified.*

### Metal Resistance Genes Annotated in the Bacterial Genome

The antibacterial biocide and metal resistance-encoding gene database (BacMet) was used to predict the presence of metal-resistance genes encoded on the genome of *S*. Typhimurium WW012. Twenty-eight resistance genes related to arsenic, copper, iron, zinc, magnesium, cobalt, mercury, molybdenum, nickel, and silver, were located on the chromosome, whilst 7 tellurium resistance encoding genes were identified as *terA/B/C/D/E/W/Z* (**Supplementary Figure S2** and **Supplementary Table S3**) on plasmid pWW012.

### Comparative Analysis of Plasmid pWW012

The *mcr-1* gene located on plasmid pWW012 was bracketed by two IS*30* elements that were located in the same orientation. Similar structures were found in plasmids pHYEC7-*mcr-1* (KX518745), pHNSHP45-2 (KU341381), and pSCC4 (CP021078) recovered previously from *E. coli* and *Citrobacter braakii*, respectively, (**Supplementary Figure S3A**). The insertion element IS*30* showed 100% (95% query coverage) of homologous sequence with IS*Apl1* in plasmid pHNSHP45-2, where in 17 amino acids were missing at the 5′-end comparing with the latter (**Supplementary Figure S3A**). In addition to the *mcr-1* gene locus, plasmid pWW012 also contained an additional MDR gene cassette embedded in a complex class 1 integron of 15.2-kbp (**Supplementary Figure S3B**) bracketed by two IS*26* elements in but found in inverted orientations directions, at a distance from the *mcr-1* gene locus (**Supplementary Figure S2**). The class 1 integron was preceded by a copy of insertion element IS*26*, and two transposase-encoding genes *tnpR* and *tnpM*, followed by the *aadA2, cmlA1*, and *aadA1* genes. Downstream of these resistance genes, an interesting resistance gene locus consisting of IS*406*–*sul3*-*orf*-*orf*-*orf*-IS*26* was identified. This locus was followed by one integrase, two resistance genes of *dfrA12* and *aadA2*, and the IS*26* element. This structure has also been identified in two *E. coli* originated plasmids designated as pHNLDF400 (KV019258.1) and pHNSHP45-2 (KU341381) (**Supplementary Figure S3B**).

## Discussion

In the past, *Salmonella* has attracted much attention owing to its important role as a food-borne pathogen. Recent studies reported on the prevalence of *Salmonella* and plasmid-encoded polymyxin resistance genes, containing *mcr-1*, among humans, food-producing animals and food samples in China and elsewhere (Anjum et al., 2016; Campos et al., 2016; Doumith et al., 2016; El Garch et al., 2016; Figueiredo et al., 2016; Kempf et al., 2016; Liu et al., 2016; Quesada et al., 2016; Yang et al., 2016). However, limited comprehensive epidemiological data are available describing the prevalence of *Salmonella* and *mcr-1* gene among RTE pork samples in China, although pork and pork-related products are the main source of animal protein for Chinese consumers. To the best of our knowledge, the current study was the first report on the epidemiological prevalence and detection of *Salmonella* and *mcr-1* gene among RTE pork samples in China. Generally, the prevalence rate of *Salmonella* and the *mcr-1* gene among RTE pork samples was relatively low in this study, while similar results were observed from clinical, pigs and retail pork in previous studies (Figueiredo et al., 2016; Quesada et al., 2016; Cui et al., 2017). However, considering the fact that these RTE pork samples are positive for *Salmonella* and therefore would not be heated or cooked the infectious risk would increase. In China, only prepackaged RTE foodstuffs have a pathogen limiting standard applied at National level. In this study all RTE pork samples were collected from marketing sites and nearly 87% of the samples that were positive for *Salmonella* were unpackaged. Although these pork samples were permitted for sale by the food hygiene bureau at the beginning, unpacked treatment may later increase the probability of the contamination of *Salmonella* during their shelf-life. Therefore, limited standard and effective legislation in RTE pork to prevent and control the contamination of *Salmonella* should now be considered.

In this study, we successfully recovered and characterized one *Salmonella* isolate (*S*. Typhimurium WW012) that harbored a *mcr-1*-bearing plasmid from a RTE prepared pork sample in China. The genus *Salmonella* consists of a number of serovars, where in it is reported that the most often detected serovar harboring the *mcr-1*-was Typhimurium (Doumith et al., 2016; Cui et al., 2017), suggesting that the acquisition and prevalence of *mcr-1*-bearing mobile elements may require a specific genetic background. The relationship between *S.* Typhimurium and *mcr-1*-bearing plasmids warrants further investigation (Doumith et al., 2016). This RTE pork isolate expressed a MDR phenotype in addition to colistin. Notably, all of the resistant antimicrobial agents tested in this study are widely used at human and veterinary clinics. Carnevali et al. (2016) screened for the *mcr-1* gene in 4,473 *Salmonella* isolates from human, food-producing animal, food and environment samples and identified 25 positive isolates of human and veterinary origin, all of which were susceptible to broad-spectrum cephalosporins. The *S*. Typhimurium WW012 isolate in this study was also susceptible to all tested cephalosporin antimicrobials. Similar results were reported from human, food-producing animals in China (Li et al., 2016; Yi et al., 2017).

A laboratory-based conjugation assay revealed that the *mcr-1* gene was transferrable to the plasmid-free recipient *E. coli* J53, with a frequency of 1.2 × 10^-6^. In contrast, the transfer frequency of the IncI2-type plasmid carrying the *mcr-1* gene reported in the original study was surprisingly higher, ranging from 10^-1^ to 10^-3^ between *E. coli* strains (Liu et al., 2016). In this study these data showed that the transfer of the *mcr-1* gene from *S*. Typhimurium WW012 to *E. coli* had a frequency that was lower. Nonetheless, it was confirmed that the *mcr-1*-carrying IncHI2-type plasmid in this study can be transferred between different genera of Enterobacteriaceae. Of note, according to previous reports, the IncHI2 replicon was one of the most common found in *Salmonella* (Campos et al., 2016; Doumith et al., 2016). Moreover, several reports confirmed *mcr-1*-positive *Salmonella* that were expressing a MDR phenotype (Doumith et al., 2016; Figueiredo et al., 2016; Yang et al., 2016). Notably, the current data showed that this transconjugant showed resistance to five antimicrobial agents in addition to colistin, indicating that *S*. Typhimurium WW012 could not only transfer the *mcr-1* gene but also other MDR-encoding genes into other Enterobacteriaceae strains. Therefore, once this clone carrying *mcr-1* gene disseminates from RTE pork to humans via the food chain, it could be expected to promote the forward dissemination of the latter marker and other members of the Enterobacteriaceae. In this study *S*. Typhimurium WW012 belonged to ST34, which is the predominant sequence type in southern China (Sun et al., 2014). Recently, the *mcr-1* gene was also detected in MDR *S.* Typhimurium (ST34) in China and Europe (Doumith et al., 2016; Li et al., 2016).

In this study, the plasmid mediated colistin resistance gene *mcr-1* was found to be located in a composite transposon with the structure IS*30*-*mcr-1*-*orf*-*orf*-IS*30*, which was similar to those found previously on two *E. coli* plasmids pHYEC7-*mcr-1* (KX518745) and pHNSHP45-2 (KU341381), and one *C. braakii* plasmid pSCC4 (CP021078, unpublished) (Liu et al., 2016; Wang et al., 2018). Unlike plasmid pHNSHP45-2, the original IncHI2-type *mcr-1-*carrying episome, the *mcr-1* gene located on plasmids pWW012, pHYEC7-*mcr-1* and pSCC4 was bracketed by IS*30* elements. According to a latest report, IS*Apl1* is a member of the IS*30* family of insertion sequences, which utilize a ‘copy-out, paste-in’ mechanism with a targeted transposition pathway requiring the formation of a synaptic complex between an inverted repeat (IR) in the transposon circle and an IR-like sequence in the target (Wang et al., 2018). IS*30* is related to site-specific recombination, which could mediate the transmission of *mcr-1* among different bacterial species. Moreover, it was found that several resistance genes were also found to be present and located between two IS*26* elements on plasmid pWW012, in which a class 1 integron similar to that found in plasmids pHNSHP45-2 and pHNLDF400 (KY019258.1) also existed (Liu et al., 2016; Wang et al., 2017). The recovery of plasmid pWW012 that harbored structurally similar mobile elements to these contained in these reported plasmids implied that genetic exchange between such plasmids was common, and that such plasmids could readily be transferred to other bacteria, constituting one major evolution and resistance development route for bacterial pathogens.

In addition to the plasmid-mediated colistin resistance gene *mcr-1*, genes found on chromosome containing mutations, encoding two component regulatory systems (*pmrAB, phoPQ*), were also found in this study. These chromosomal mutations mediated mechanisms were thought to lead to the modification of lipid A, which anchors the lipopolysaccharide molecule to the outer membrane, resulting in reduction in the affinity to colistin (Lee et al., 2014). Moreover, a target gene mutation in the quinolone resistance determining region (QRDR) on the chromosome were identified in *gyrA* mutation (giving rise to D87N amino acid substitution) in this *mcr-1* harboring *S*. Typhimurium. The latter is frequently reported in other MDR *Salmonella* species in China (Wong et al., 2014). In addition, *strA/B* encoding streptomycin resistance and *sul2* encoding sulfamethoxazole resistances were also detected to locate on the chromosome of *S*. Typhimurium WW012.

In this study, 201 virulence genes were found on the chromosome of *S*. Typhimurium WW012 when queried through VFDB. Type-three secretion systems (T3SS) encoding genes related to SPI-1 and SPI-2 were commonly detected. Both SPI-1 and SPI-2 contained two independent type three secretion systems (denoted as TTSS SPI-1 and TTSS SPI-2, respectively) that can inject effect or proteins into host cells, which are crucial for various stages of infection (Weening et al., 2005). Comparison of the genomes *S*. Typhimurium WW012 with the reference or complete sequences in NCBI for the presence of SPIs showed that SPIs in *S*. Typhimurium WW012 were closely related to their equivalents in *S*. Typhimurium LT2 (McClelland et al., 2001), and shared some of those in *S*. Typhi CT18 and *S*. Indiana C629 (Parkhill et al., 2001; Wang et al., 2016). Additionally, 63 fimbrial adherence encoding genes on the chromosome of *S*. Typhimurium WW012 were also identified in this study. Several *in vitro* studies have suggested that fimbrial-encoding genes found in *Salmonella* could mediate the attachment of this bacterium to epithelial cells in the host (Leclerc et al., 2016; Peters et al., 2017). The latter phenotype may support long-term intestinal carriage of this bacterium.

Heavy metal compounds as well as antimicrobial agents are widely used as feed additives in food-producing animals for therapy, and growth purposes in China (Hu et al., 2017). Because of their stable and persistent characteristics, when heavy metals accumulate to specific concentrations, they potentially result in resistance among bacteria cultured from food-producing animals. Heavy metal resistance-encoding genes have also been identified in different environments (Deng et al., 2017). Recent research data showed that a relationship between the acquisition of heavy metal resistance genes and antimicrobial resistance genes, and antimicrobial resistance may arise through co-resistance or cross-resistance to metals or co-regulation of resistance pathways (Argudín et al., 2016; Deng et al., 2017). In this study, 28 and 7 heavy metal resistance genes were found on the chromosome and plasmid, respectively. These heavy metal resistance genes might increase antimicrobial resistance capacity within this bacterium, through co-selection of determinants.

## Conclusion

This study firstly reported the epidemiological prevalence and detection of *Salmonella* and *mcr-1* gene among RTE pork samples in China. These data highlight the importance of role played by *S*. Typhimurium in the dissemination of MDR genes. Although the mechanisms remain to be further described, the emergence of MDR genes particularly *mcr-1*, along with various virulence factors and heavy metal resistance genes, on the chromosome and plasmid from *S*. Typhimurium, will challenge therapeutic options for clinicians and others. Moreover, successful transmission of the *mcr-1* gene poses a challenge to those with an interest in the protection of public health.

## Availability of Data and Materials

The aggregate data supporting findings contained within this manuscript will be shared upon request submitted to the corresponding author.

## Author Contributions

WW, ZB, SF, and FL designed the experiments and wrote the manuscript. MZ, YD, YH, ZP, and JX carried out the experiments. ZB, WW, and NY analyzed the experimental results.

## Conflict of Interest Statement

The authors declare that the research was conducted in the absence of any commercial or financial relationships that could be construed as a potential conflict of interest. The reviewer MS and handling Editor declared their shared affiliation.

## Footnotes

1 http://rast.nmpdr.org

2 http://blast.ncbi.nlm.nih.gov/Blast.cgi

3 http://www.ncbi.nlm.nih.gov/orffinder

4 https://cge.cbs.dtu.dk/services/PlasmidFinder/

5 https://card.mcmaster.ca/analyze

6 http://www.mgc.ac.cn/VFs/main.htm

7 http://cge.cbs.dtu.dk/services/SPIFinder

8 http://bacmet.biomedicine.gu.se/

## Supplementary Material

FIGURE S1 S1-PFGE indicating the transfer of single plasmid harboring *mcr-1*. Lane 1, *Escherichia coli* J53 (recipient); Lane 2, *S.* Typhimurium WW012 (donor); Lane 3, CT-*E. coli* J53(transconjugant); Lane 4, H-9812(PFGE marker strain, *S*.BraenderupH9812).

FIGURE S2 A schematic representation of the complete chromosome **(A)** and plasmid **(B)** of *S.* Typhimurium WW012. Each ring, beginning from the inside to out represents the following features: GC content (black); GC skew (green and purple); CDS (blue); Antimicrobial resistance genes (shown in blue colored font), metal resistance genes (shown in green colored font), and virulence factor genes (shown in black colored font).

FIGURE S3 Major structural features of plasmid pWW012 compared with several other plasmids identified from NCBI. **(A)** Major structural features of plasmid pWW012 compared with plasmids pHYEC7-mcr-1, pSCC4, and pHNSHP45-2 identified in *E. coli* and *Citrobacter braakii*, respectively. **(B)** Major structural features of plasmid pWW012 compared with plasmids pHNLDF400 and pHNSHP45-2 both identified in *E. coli*. ORFs are shown with arrows (blue arrows, transposase; red arrows, antibiotic resistance genes; green arrows, integrase; gray arrows, member protein; white arrows, hypothetical protein–see the key to the right hand side of the panel). The missing amino acids in IS*30* comparing with IS*Apl1* were showed in red box.

TABLE S1 Sampling information of *Salmonella* isolates in this study.

TABLE S2 Virulence resistance-encoding genes identified in *S*. Typhimurium WW012 when comparing the genome of the latter against the current version of the VFDB database.

TABLE S3 Metal resistance genes of *S*. Typhimurium WW012 with the current version of the BacMet database.
