# Supplementary material for: Complete Genomic Analysis of a Salmonella enterica Serovar Typhimurium Isolate Cultured From Ready-to-Eat Pork in China Carrying One Large Plasmid Containing mcr-1
Source: Front Microbiol. 2018 Apr 27;9:616. doi: 10.3389/fmicb.2018.00616 (PMC5934421; doi:10.3389/fmicb.2018.00616)

**Supplemental Table 1 Sampling information of *Salmonella* isolates in this study**

| **Isolate** | **Serotype** | **Sources** | **Packing type** | **Amount** | **Date of Sampling** | **Province** | **Sampling site** | **Manufacturers** | **Date of manufacture** |
| --- | --- | --- | --- | --- | --- | --- | --- | --- | --- |
| S.1 | Typhimurium | Pork chops | unpacked | >500g | 2014.01.05 | Jiangsu | commercial hypermarkets | Shanghai Phoenix foods limited | 2014.01.05 |
| S.2 | Derby | Pig Feet Seasoned with Soy Sauce | unpacked | >500g | 2014.10.22 | Jiangsu | commercial hypermarkets | Shanghai da-Mu Khan Foods Ltd | 2014.10.22 |
| S.3 | Derby | Pig Ear in Sauce | unpacked | >500g | 2014.05.18 | Henan | retail outlets | Szhou food co | 2014.05.18 |
| S.4 | Infantis | pork sausage | unpacked | >500g | 2014.05.18 | Henan | retail outlets | Szhou food co | 2014.05.18 |
| S.5 | Uganda | prepared pork | unpacked | >500g | 2014.04.27 | Henan | retail outlets | Unknown | 2014.04.27 |
| S.6 | Derby | pork ball | unpacked | >500g | 2014.10.12 | Zhejiang | retail outlets | Unknown | 2014.10.12 |
| S.7 | Derby | Marinated pork feets | unpacked | >500g | 2014.05.11 | Zhejiang | retail outlets | Liu Yang 18055443274 | 2014.05.11 |
| S.8 | Enteritidis | Marinated black pepper pork chop | unpacked | >500g | 2014.05.11 | Fujian | retail outlets | Pang Shiwu 13695543126 | 2014.05.11 |
| S.9 | Derby | Chinese salad with pork meat | unpacked | >500g | 2014.10.12 | Jiangxi | retail outlets | Unknown | 2014.10.12 |
| S.10 | Reading | Sliced Pork in Soy Sauce | unpacked | >500g | 2014.09.12 | Sichuan | retail outlets | Unknown | 2014.09.12 |
| S.11 | Reading | Marinated black pepper pork chop | unpacked | >500g | 2014.05.11 | Sichuan | retail outlets | Wu li 1855344474 | 2014.05.11 |
| *S*. Typhimurium WW012 | Typhimurium | prepared porkthat is ready-to-eat | unpacked | >500g | 2014.09.14 | Guangxi | retail outlets | Convenience market | 2014.09.14 |
| S.13 | Typhimurium | Roast Suckling Pig | unpacked | >500g | 2014.05.11 | Guangxi | retail outlets | Ominous | 2014.05.11 |
| S.14 | Corvallis | Marinated Pig Tongue | unpacked | >500g | 2014.05.12 | Beijing | retail outlets | Shandong linyi Lu Cheng food co | 2014.05.12 |
| S.15 | Derby | Cooking pork | prepackage | >500g | 2014.05.13 | Jilin | retail outlets | Xin sheng, Gaoping meat co | 2014.05.13 |
| S.16 | Derby | Cooking pork | unpacked | >500g | 2014.10.12 | Shanxi | retail outlets | Shanxi Changzhi West Street butcher's shop | 2014.10.12 |
| S.17 | Derby | Cooking pork offal | unpacked | >500g | 2014.10.12 | Shanxi | retail outlets | Gulou Street, licheng into a butcher's shop | 2014.10.12 |
| S.18 | London | Spicy Pork Ribs | unpacked | >500g | 2014.10.12 | Anhui | retail outlets | Unknown | 2014.10.12 |
| S.19 | Enteritidis | Pork with Garlic Sauce | unpacked | >500g | 2014.8.10 | Anhui | retail outlets | Unknown | 2014.8.10 |
| S.20 | Agona | Marinated pork feets | unpacked | >500g | 2014.10.12 | Shanghai | retail outlets | Unknown | 2014.10.12 |
| S.21 | Rissen | pork ball | prepackage | >500g | 2014.10.12 | Shanghai | retail outlets | Unknown | 2014.10.12 |
| S.22 | Typhimurium | Pig Ear in Sauce | unpacked | >500g | 2014.10.12 | Guangdong | retail outlets | Unknown | 2014.10.12 |
| S.23 | Typhimurium | Marinated Pig Tongue | unpacked | >500g | 2014.10.11 | Guangdong | retail outlets | Unknown | 2014.10.11 |
| S.24 | Typhimurium | Pork chops | unpacked | >500g | 2014.8.10 | Chongqing | retail outlets | Unknown | 2014.8.10 |
| S.25 | Enteritidis | pork head meat | prepackage | >500g | 2014.10.12 | Hubei | commercial hypermarkets | Noble, Chaoyang Street, shouyang Times Square Shopping Center | 2014.10.12 |
| S.26 | Enteritidis | Pork chops | unpacked | >500g | 2014.10.12 | Hubei | retail outlets | the pork shop | 2014.10.12 |
| S.27 | Enteritidis | Pig Ear in Sauce | unpacked | >500g | 2014.04.08 | Hunan | commercial hypermarkets | Noble times square every good supermarket | 2014.04.08 |
| S.28 | Enteritidis | pork sausage | prepackage | >500g | 2014.04.08 | Xinjiang | retail outlets | Convenience market | 2014.04.08 |
| S.29 | Uganda | Marinated pork feets | unpacked | >500g | 2014.09.09 | Shandong | retail outlets | street market | 2014.09.09 |
| S.30 | Uganda | Pork head meat | unpacked | >500g | 2014.05.11 | Shandong | retail outlets | Shandong liuhe group Xiaoyi elephant farm foods limited | 2014.05.11 |

**Supplemental Table 2 Virulence resistance-encoding genes identified in S. TyphimuriumWW012 when comparing the genome of the latter against the current version of the VFDB database**

| **gene** | **Decription** | **Virulence factors** |
| --- | --- | --- |
| *sinH* | Aec1 | Aec1 |
| *shdA* | AIDA autotransporter-like protein | AIDA autotransporter-like protein |
| *rpoS* | sigma S (sigma 38) factor of RNA polymerase, major sigmafactor during stationary phase | Alternative sigma factor RpoS |
| [*mig-14*](http://www.mgc.ac.cn/cgi-bin/VFs/vfs.cgi?VFID=VF0321#VF0321) | antimicrobial peptide resistance protein Mig-14 | antimicrobial peptide resistance protein Mig-14 |
| [*csgA*](http://www.mgc.ac.cn/cgi-bin/VFs/vfs.cgi?Genus=Salmonella&Keyword=Adherence) | curlin major subunit CsgA | Fimbrial adherence determinants |
| [*csgC*](http://www.mgc.ac.cn/cgi-bin/VFs/vfs.cgi?VFID=VF0103#VF0103) | curli assembly protein CsgC | Fimbrial adherence determinants |
| [*csgE*](http://www.mgc.ac.cn/cgi-bin/VFs/vfs.cgi?VFID=VF0105#VF0105) | curli production assembly/transport protein CsgE | Fimbrial adherence determinants |
| [*csgG*](http://www.mgc.ac.cn/cgi-bin/VFs/vfs.cgi?VFID=VF0397#VF0397) | curli production assembly/transport protein CsgG | Fimbrial adherence determinants |
| [*csgB*](http://www.mgc.ac.cn/cgi-bin/VFs/vfs.cgi?VFID=VF0104#VF0104) | nucleation component of curlin monomers | Fimbrial adherence determinants |
| [*csgD*](http://www.mgc.ac.cn/cgi-bin/VFs/vfs.cgi?VFID=VF0399#VF0399) | DNA-binding transcriptional regulator CsgD | Fimbrial adherence determinants |
| [*bcfD*](http://www.mgc.ac.cn/cgi-bin/VFs/vfs.cgi?VFID=VF0400#VF0400) | hypothetical protein | Fimbrial adherence determinants |
| [*bcfB*](http://www.mgc.ac.cn/cgi-bin/VFs/vfs.cgi?VFID=VF0102#VF0102) | fimbrialchaparone | Fimbrial adherence determinants |
| [*bcfC*](http://www.mgc.ac.cn/cgi-bin/VFs/vfs.cgi?Genus=Salmonella&Keyword=Antivirulence) | fimbrial usher | Fimbrial adherence determinants |
| [*bcfE*](http://www.mgc.ac.cn/cgi-bin/VFs/vfs.cgi?VFID=VF0110#VF0110) | fimbrial subunit | Fimbrial adherence determinants |
| [*bcfF*](http://www.mgc.ac.cn/cgi-bin/VFs/vfs.cgi?Genus=Salmonella&Keyword=Immune%20evasion) | fimbrial subunit | Fimbrial adherence determinants |
| [*bcfG*](http://www.mgc.ac.cn/cgi-bin/VFs/vfs.cgi?VFID=VF0101#VF0101) | fimbrialchaparone | Fimbrial adherence determinants |
| [*fimA*](http://www.mgc.ac.cn/cgi-bin/VFs/vfs.cgi?Genus=Salmonella&Keyword=Magnesium%20uptake) | type-1 fimbrial protein, A chain | Fimbrial adherence determinants |
| [*fimC*](http://www.mgc.ac.cn/cgi-bin/VFs/vfs.cgi?VFID=VF0106#VF0106) | fimbrial chaperone protein | Fimbrial adherence determinants |
| [*fur*](http://www.mgc.ac.cn/cgi-bin/VFs/vfs.cgi?Genus=Salmonella&Keyword=Regulation) | transcriptional repressor of iron-responsive genes (Fur family) (ferric uptake regulator) | Fimbrial adherence determinants |
| [*ompD*](http://www.mgc.ac.cn/cgi-bin/VFs/vfs.cgi?VFID=VF0113#VF0113) | outer membrane porin precursor | Fimbrial adherence determinants |
| [*lpfB*](http://www.mgc.ac.cn/cgi-bin/VFs/vfs.cgi?VFID=VF0111#VF0111) | long polar fimbrial chaperone protein LpfB | Fimbrial adherence determinants |
| [*lpfC*](http://www.mgc.ac.cn/cgi-bin/VFs/vfs.cgi?VFID=VF0112#VF0112) | long polar fimbrial usher protein LpfC | Fimbrial adherence determinants |
| [*lpfD*](http://www.mgc.ac.cn/cgi-bin/VFs/vfs.cgi?Genus=Salmonella&Keyword=Resistance%20to%20antimicrobial%20peptides) | long polar fimbrial protein LpfD | Fimbrial adherence determinants |
| [*lpfE*](http://www.mgc.ac.cn/cgi-bin/VFs/vfs.cgi?VFID=VF0395#VF0395) | long polar fimbrial minor subunit LpfE, adhesin | Fimbrial adherence determinants |
| [*misL*](http://www.mgc.ac.cn/cgi-bin/VFs/vfs.cgi?Genus=Salmonella&Keyword=Serum%20resistance) | putative autotransporter | Fimbrial adherence determinants |
| [*nlpI*](http://www.mgc.ac.cn/cgi-bin/VFs/vfs.cgi?VFID=VF0108#VF0108) | lipoprotein NlpI | Fimbrial adherence determinants |
| *ratB* | RatB | Fimbrial adherence determinants |
| *pagN* | adhesin/invasin protein PagN | Fimbrial adherence determinants |
| *stfA* | putative fimbrial subunit | Fimbrial adherence determinants |
| *stfC* | putative fimbrial outer membrane usher | Fimbrial adherence determinants |
| *stfD* | putative periplasmicfimbrial chaperone | Fimbrial adherence determinants |
| *stfE* | putative minor fimbrial subunit | Fimbrial adherence determinants |
| *stfF* | putative minor fimbrial subunit | Fimbrial adherence determinants |
| *stfG* | putative minor fimbrial subunit | Fimbrial adherence determinants |
| *safB* | putative fimbriae assembly chaparone | Fimbrial adherence determinants |
| *safD* | putative fimbrial structural subunit | Fimbrial adherence determinants |
| *safC* | putative fimbrial usher | Fimbrial adherence determinants |
| *stbA* | putative fimbrial major subunit | Fimbrial adherence determinants |
| *stbB* | putative fimbrial chaperone | Fimbrial adherence determinants |
| *stbC* | putative fimbrial usher | Fimbrial adherence determinants |
| *stbD* | putative fimbrial usher | Fimbrial adherence determinants |
| *stcA* | putative fimbrial-like protein | Fimbrial adherence determinants |
| *stcB* | putative periplasmic chaperone protein | Fimbrial adherence determinants |
| *stcC* | putative outer membrane protein | Fimbrial adherence determinants |
| *stcD* | putative outer membrane lipoprotein | Fimbrial adherence determinants |
| *stdC* | putative fimbrialchaparone | Fimbrial adherence determinants |
| *stdD* | outer membrane protein | Fimbrial adherence determinants |
| *stdA* | putative fimbrial-like protein | Fimbrial adherence determinants |
| *stdB* | fimbrial usher protein | Fimbrial adherence determinants |
| *sthA* | putative fimbrialchaparone | Fimbrial adherence determinants |
| *sthB* | putative fimbrialchaparone | Fimbrial adherence determinants |
| *sthC* | Outer membrane fimbrial usher protein | Fimbrial adherence determinants |
| *sthE* | putative fimbrial subunit | Fimbrial adherence determinants |
| *stiA* | putative fimbrial subunit | Fimbrial adherence determinants |
| *stiB* | putative fimbrialchaparone | Fimbrial adherence determinants |
| *stiC* | putativiefimbrial usher | Fimbrial adherence determinants |
| *stiH* | putative fimbrial protein precurosr | Fimbrial adherence determinants |
| *stjA* | putative outer membrane protein | Fimbrial adherence determinants |
| *stjB* | putative fimbrial usher protein | Fimbrial adherence determinants |
| *stjC* | putative fimbrialchaparone | Fimbrial adherence determinants |
| *STM4575* | putative outer membrane protein | Fimbrial adherence determinants |
| *fimI* | fimbrial protein internal segment | Fimbrial adherence determinants |
| *fimD* | usher protein FimD | Fimbrial adherence determinants |
| *fimH* | type I fimbriae minor fimbrial subunit FimH, adhesin | Fimbrial adherence determinants |
| *fimW* | putative fimbrial protein | Fimbrial adherence determinants |
| *fimY* | putative regulatory protein | Fimbrial adherence determinants |
| *fimZ* | fimbrial protein Z | Fimbrial adherence determinants |
| [*flgJ*](http://www.mgc.ac.cn/cgi-bin/VFs/vfs.cgi?Genus=Salmonella&Keyword=Stress%20protein) | flagellar rod assembly protein/muramidaseFlgJ | Flagella |
| [*flgB*](http://www.mgc.ac.cn/cgi-bin/VFs/vfs.cgi?VFID=VF0109#VF0109) | flagellar basal body rod protein FlgB | Flagella |
| [*flgC*](http://www.mgc.ac.cn/cgi-bin/VFs/vfs.cgi?Genus=Salmonella&Keyword=Toxin) | flagellar basal body rod protein FlgC | Flagella |
| [*flgF*](http://www.mgc.ac.cn/cgi-bin/VFs/vfs.cgi?VFID=VF0407#VF0407) | flagellar basal body rod protein FlgF | Flagella |
| [*flgG*](http://www.mgc.ac.cn/cgi-bin/VFs/vfs.cgi?VFID=VF0107#VF0107) | flagellar basal body rod protein FlgG | Flagella |
| [*cheA*](http://www.mgc.ac.cn/cgi-bin/VFs/vfs.cgi?Genus=Salmonella&Keyword=Unclassified) | chemotaxis protein CheA | Flagella |
| [*cheB*](http://www.mgc.ac.cn/cgi-bin/VFs/vfs.cgi?VFID=VF0396#VF0396) | chemotaxis-specific methylesterase | Flagella |
| *cheR* | chemotaxismethyltransferaseCheR | Flagella |
| [*cheW*](http://www.mgc.ac.cn/cgi-bin/VFs/pai.cgi?Genus=Salmonella&PAI=SPI-1) | purine-binding chemotaxis protein | Flagella |
| [*cheZ*](http://www.mgc.ac.cn/cgi-bin/VFs/pai.cgi?Genus=Salmonella&PAI=SPI-2) | chemotaxis regulator CheZ | Flagella |
| [*flgA*](http://www.mgc.ac.cn/cgi-bin/VFs/pai.cgi?Genus=Salmonella&PAI=SPI-3) | flagellar basal body P-ring biosynthesis protein FlgA | Flagella |
| [*flgH*](http://www.mgc.ac.cn/cgi-bin/VFs/pai.cgi?Genus=Salmonella&PAI=SPI-4) | flagellar basal body L-ring protein | Flagella |
| [*flgI*](http://www.mgc.ac.cn/cgi-bin/VFs/pai.cgi?Genus=Salmonella&PAI=SPI-5) | flagellar basal body P-ring protein | Flagella |
| *flgL* | flagellar hook-associated protein FlgL | Flagella |
| *flgN* | FlgK/FlgL export chaperone | Flagella |
| *flhC* | transcriptional activator FlhC | Flagella |
| *flhD* | transcriptional activator FlhD | Flagella |
| *flhE* | flagellar protein | Flagella |
| *fliA* | flagellar biosynthesis sigma factor | Flagella |
| *fliB* | lysine-N-methylase | Flagella |
| *fliC* | flagellin | Flagella |
| *fliD* | flagellar capping protein | Flagella |
| *fliE* | flagellar hook-basal body protein FliE | Flagella |
| *fliG* | flagellar motor switch protein G | Flagella |
| *fliI* | flagellum-specific ATP synthase | Flagella |
| *fliJ* | flagellar biosynthesis chaperone | Flagella |
| *fliL* | flagellar basal body-associated protein FliL | Flagella |
| *fliM* | flagellar motor switch protein FliM | Flagella |
| *fliN* | flagellar motor switch protein FliN | Flagella |
| *fliP* | flagellar biosynthesis protein FliP | Flagella |
| *fliR* | flagellar biosynthesis protein FliR | Flagella |
| *fliS* | flagellar protein FliS | Flagella |
| *fliT* | flagellar biosynthesis protein FliT | Flagella |
| *fliY* | cystine transporter subunit | Flagella |
| *fliZ* | flagella biosynthesis protein FliZ | Flagella |
| *flk* | flagella biosynthesis regulator | Flagella |
| *motA* | flagellar motor protein MotA | Flagella |
| *motB* | flagellar motor protein MotB | Flagella |
| *sodCI* | Gifsy-2 prophage: superoxide dismutase precursor (Cu-Zn) | Gifsy-2 prophage: superoxide dismutase precursor |
| [*siiE*](http://www.mgc.ac.cn/cgi-bin/VFs/vfs.cgi?VFID=VF0398#VF0398) | inner membrane protein | Inner membrane protein |
| [*mgtB*](http://www.mgc.ac.cn/cgi-bin/VFs/vfs.cgi?Genus=Salmonella&Keyword=Secretion%20system) | Mg2+ transport protein | Mg2+ transport protein |
| [*mgtC*](http://www.mgc.ac.cn/cgi-bin/VFs/vfs.cgi?VFID=VF0116#VF0116) | Mg2+ transport protein | Mg2+ transport protein |
| *iroB* | glycosyltransferase | Salmochelin synthesis and transport |
| *iroC* | ABC transporter protein | Salmochelin synthesis and transport |
| *iroD* | enterochelin esterase=-like protein | Salmochelin synthesis and transport |
| *iroE* | hydrolase | Salmochelin synthesis and transport |
| *iroN* | outer membrane receptor FepA | Salmochelin synthesis and transport |
| *sciS* | inner membrane protein | SCI (Salmonella centrisome island) |
| *STM0278* | periplasmic protein | SCI (Salmonella centrisome island) |
| *clpV* | chaperone ATPase | SCI (Salmonella centrisome island) |
| *sciB* | cytoplasmic protein | SCI (Salmonella centrisome island) |
| *sciC* | cytoplasmic protein | SCI (Salmonella centrisome island) |
| *sciD* | cytoplasmic protein | SCI (Salmonella centrisome island) |
| *sciE* | cytoplasmic protein | SCI (Salmonella centrisome island) |
| *sciF* | cytoplasmic protein | SCI (Salmonella centrisome island) |
| *sciH* | cytoplasmic protein | SCI (Salmonella centrisome island) |
| *sciI* | cytoplasmic protein | SCI (Salmonella centrisome island) |
| *sciJ* | cytoplasmic protein | SCI (Salmonella centrisome island) |
| *sciK* | cytoplasmic protein | SCI (Salmonella centrisome island) |
| *sciL* | cytoplasmic protein | SCI (Salmonella centrisome island) |
| *sciM* | cytoplasmic protein | SCI (Salmonella centrisome island) |
| *sciN* | outer membrane lipoprotein | SCI (Salmonella centrisome island) |
| *sciO* | cytoplasmic protein | SCI (Salmonella centrisome island) |
| *sciQ* | inner membrane protein | SCI (Salmonella centrisome island) |
| *sciR* | Shiga-like toxin A subunit | SCI (Salmonella centrisome island) |
| *sciT* | cytoplasmic protein | SCI (Salmonella centrisome island) |
| *sciV* | cytoplasmic protein | SCI (Salmonella centrisome island) |
| *sciW* | cytoplasmic protein | SCI (Salmonella centrisome island) |
| *sseI* | putative type III secreted protein | Secretion system (TTSS-2 translocated effectors) |
| *gogB* | leucine-rich repeat protein | Secretion system (TTSS-2 translocated effectors) |
| *sseL* | hypothetical protein | Secretion system (TTSS-2 translocated effectors) |
| *orgC* | type III secretion system effector OrgC | Secretion system [TTSS (SPI-1 encode)] |
| *steA* | type III secretion system effector SteA | Secretion system [TTSS (SPI-1 encode)] |
| *hilA* | invasion protein regulator | Secretion system [TTSS (SPI-1 encode)] |
| *invF* | hypothetical protein | Secretion system [TTSS (SPI-1 encode)] |
| *prgH* | cell invasion protein | Secretion system [TTSS (SPI-1 encode)] |
| *prgJ* | type III secretion system inner rod protein PrgJ | Secretion system [TTSS (SPI-1 encode)] |
| *avrA* | type III secretion system effector AvrA, acetyltransferease | Secretion system [TTSS (SPI-1 encode)] |
| *hilC* | invasion regulatory protein | Secretion system [TTSS (SPI-1 encode)] |
| *hilD* | invasion protein regulatory protein | Secretion system [TTSS (SPI-1 encode)] |
| *iacP* | acyl carrier protein | Secretion system [TTSS (SPI-1 encode)] |
| *iagB* | invasion protein precursor | Secretion system [TTSS (SPI-1 encode)] |
| *invB* | type III secretion system protein InvB | Secretion system [TTSS (SPI-1 encode)] |
| *invC* | type III secretion system ATPase InvC | Secretion system [TTSS (SPI-1 encode)] |
| *invG* | type III secretion system secretin invG | Secretion system [TTSS (SPI-1 encode)] |
| *invJ* | type III secretion system needle length regulator InvJ | Secretion system [TTSS (SPI-1 encode)] |
| *orgB* | type III secretion system stator OrgB | Secretion system [TTSS (SPI-1 encode)] |
| *prgI* | type III secretion system needle filament protein PrgI | Secretion system [TTSS (SPI-1 encode)] |
| *prgK* | type III secretion system inner MS ring protein PrgK | Secretion system [TTSS (SPI-1 encode)] |
| *sicA* | chaparone for SipC and SipB | Secretion system [TTSS (SPI-1 encode)] |
| *sicP* | chaparone for SptP | Secretion system [TTSS (SPI-1 encode)] |
| *sipA* | type III secretion system effector SipA, actin polymerizing activity | Secretion system [TTSS (SPI-1 encode)] |
| *sipC* | type III secretion system hydrophilic translocator, pore protein SipC | Secretion system [TTSS (SPI-1 encode)] |
| *sipD* | type III secretion system hydrophilic translocator, needle tip protein SipD | Secretion system [TTSS (SPI-1 encode)] |
| *slrP* | type III secretion system effector SlrP, E3 ubiquitin ligase | Secretion system [TTSS (SPI-1 encode)] |
| *sopA* | type III secretion system effector SopA, E3 ubiquitin ligase | Secretion system [TTSS (SPI-1 encode)] |
| *sopB* | type III secretion system effector SopB, phosphoinositide phosphatase | Secretion system [TTSS (SPI-1 encode)] |
| *sopD* | type III secretion system effector SopD | Secretion system [TTSS (SPI-1 encode)] |
| *sopE2* | type III secretion system effector SopE2, guanine nucleotide exchange factor | Secretion system [TTSS (SPI-1 encode)] |
| *spaO* | type III secretion system C ring protein SpaO | Secretion system [TTSS (SPI-1 encode)] |
| *spaR* | type III secretion system minor export apparatus protein SpaR | Secretion system [TTSS (SPI-1 encode)] |
| *sprB* | transcriptional regulator | Secretion system [TTSS (SPI-1 encode)] |
| *sptP* | type III secretion system effector SptP, tyrosine phosphatase and GTPase-activating protein | Secretion system [TTSS (SPI-1 encode)] |
| *pipB* | type III secretion system effector PipB | Secretion system [TTSS (SPI-2 encode)] |
| *pipB2* | type III secretion system effector PipB3 | Secretion system [TTSS (SPI-2 encode)] |
| *sifB* | type III secretion system effector SifB | Secretion system [TTSS (SPI-2 encode)] |
| *sopD2* | type III secretion system effector SopD2 | Secretion system [TTSS (SPI-2 encode)] |
| *sscA* | chaperone for sseC | Secretion system [TTSS (SPI-2 encode)] |
| *sscB* | chaperone for sseF | Secretion system [TTSS (SPI-2 encode)] |
| *sseK1* | type III secretion system effector SseK1 | Secretion system [TTSS (SPI-2 encode)] |
| *sseK2* | type III secretion system effector SseK2 | Secretion system [TTSS (SPI-2 encode)] |
| *ssrA* | sensor kinase | Secretion system [TTSS (SPI-2 encode)] |
| *ssrB* | transcriptional activator | Secretion system [TTSS (SPI-2 encode)] |
| *steC* | type III secretion system effector SteC | Secretion system [TTSS (SPI-2 encode)] |
| *ssaI* | hypothetical protein | Secretion system [TTSS (SPI-2 encode)] |
| *sifA* | type III secretion system effector SifA, N-terminal SKIP-binding domain | Secretion system [TTSS (SPI-2 encode)] |
| *spiC* | type III secretion system protein SsaB | Secretion system [TTSS (SPI-2 encode)] |
| *ssaC* | type III secretion system secretin SsaC | Secretion system [TTSS (SPI-2 encode)] |
| *ssaD* | type III secretion system outer MS ring protein SsaD | Secretion system [TTSS (SPI-2 encode)] |
| *ssaG* | type III secretion system needle filament protein SsaG | Secretion system [TTSS (SPI-2 encode)] |
| *ssaJ* | type III secretion system inner MS ring protein SsaJ | Secretion system [TTSS (SPI-2 encode)] |
| *ssaK* | type III secretion system stator SsaK | Secretion system [TTSS (SPI-2 encode)] |
| *ssaL* | type III secretion system gatekeeper SsaL | Secretion system [TTSS (SPI-2 encode)] |
| *ssaM* | type III secretion system protein SsaM | Secretion system [TTSS (SPI-2 encode)] |
| *ssaN* | type III secretion system ATPase SsaN | Secretion system [TTSS (SPI-2 encode)] |
| *ssaP* | type III secretion system needle length regulator SsaP | Secretion system [TTSS (SPI-2 encode)] |
| *ssaQ* | type III secretion system C ring protein SsaQ | Secretion system [TTSS (SPI-2 encode)] |
| *ssaR* | type III secretion system minor export apparatus protein SsaR | Secretion system [TTSS (SPI-2 encode)] |
| *ssaU* | type III secretion system export apparatus switch protein SsaU | Secretion system [TTSS (SPI-2 encode)] |
| *sseA* | chaperone for sseB and sseD | Secretion system [TTSS (SPI-2 encode)] |
| *sseB* | type III secretion system effector SseB | Secretion system [TTSS (SPI-2 encode)] |
| *sseE* | type III secretion system effector SseE | Secretion system [TTSS (SPI-2 encode)] |
| *sseJ* | type III secretion system effector SseJ, glycerophospholipid:cholesterolacyltransferase | Secretion system [TTSS (SPI-2 encode)] |
| *sspH2* | type III secretion system effector SspH2, E3 ubiquitin ligase | Secretion system [TTSS (SPI-2 encode)] |

**Supplemental Table 3 Metal resistance genes of *S*. TyphimuriumWW012 with the current version of the BacMet database**

| **Gene** | **Resistance mechanism or resistant to** | **Origin strain** | **Location** |
| --- | --- | --- | --- |
| **Chromosome** | | | |
| *arsC* | Arsenate reductase | *A. multivorum* | 4445627..4446049 |
| *arsB* | Arsenical pump membrane protein | *Y. enterocolitica* | 4446065..4447351 |
| *arsA* | Arsenical pump-driving ATPase | *A. multivorum* | 4447402..4449150 |
| *arsR* | Arsenical resistance operon repressor | *E. coli* | 4449581..4449931 |
| *arsD* | Arsenical resistance operon trans-acting repressor ArsD | *E. coli* | 4449207..4449530 |
| *cueO* | Blue copper oxidase CueO | *S*. Typhimurium | 3900791..3902398 |
| *pcoA* | Copper resistance protein A | *E. coli* | 4400037..4401830 |
| *pcoB* | Copper resistance protein B | *E. coli* | 4399120..4400007 |
| *pcoC* | Copper resistance protein C | *E. coli* | 4398700..4399077 |
| *pcoD* | Copper resistance protein D | *E. coli* | 4397766..4398629 |
| *fieF* | Ferrous-iron efflux pump FieF | *E. coli* | 4764854..4765741 |
| *znuB* | High-affinity zinc uptake system membrane protein ZnuB | *E. coli* | 2117613..2118395 |
| *sitA* | Iron transport protein, periplasmic-binding protein | *S*. Typhi | 1069504..1070418 |
| *corC* | Magnesium and cobalt efflux protein CorC | *S*. Typhimurium | 3323740..3324615 |
| *corA* | Magnesium transport protein CorA | *S*. Typhimurium | 4877671..4878618 |
| *mgtA* | Magnesium-transporting ATPase, P-type 1 | *S*. Typhimurium | 4253951..4256656 |
| *merA* | Mercuric reductase (Fragments) | *Serratiamarcescens* | 1169426..1169779 |
| *merR* | Mercuric resistance operon regulatory protein | *Serratiamarcescens* | 1172338..1172754 |
| *merT* | Mercuric transport protein | *Pseudomonas* sp. | 1171919..1172266 |
| *modC* | Molybdenum import ATP-binding protein ModC | *E. coli* | 3208307..3209362 |
| *modB* | Molybdenum transport system permease protein ModB | *E. coli* | 3209368..3210054 |
| *nikR* | Nickel-responsive regulator | *E. coli* | 293888..294286 |
| *silP* | Silver exporting P-type ATPase | *S*. Typhimurium | 4403821..4406271 |
| *silE* | Silver-binding protein SilE | *S*. Typhimurium | 4415821..4416249 |
| *znuC* | Zinc import ATP-binding protein ZnuC | *E. coli* | 2118395..2119147 |
| *zraP* | Zinc resistance-associated protein | *S*.Typhimurium | 4648907..4649275 |
| *zupT* | Zinc transporter ZupT | *E. coli* | 723723..724493 |
| *zur* | Zinc uptake regulation protein | *E. coli* | 4574772..4575284 |
| **plasmid** | | | |
| *terZ* | Tellurium resistance protein TerZ | *Serratia*marcescens | 23853..24431 |
| *terD* | Tellurium resistance protein TerD | *Alcaligenes* sp. | 24480..25520 |
| *terC* | Tellurium resistance protein TerC | *Alcaligenes* sp. | 25543..25998 |
| *terB* | Tellurium resistance protein TerB | *Alcaligenes* sp. | 26021..27178 |
| *terE* | Telluriumresistance protein TerE | *Alcaligenes* sp. | 27178..27759 |
| *terA* | Tellurium resistance protein TerA | *Alcaligenes* sp. | 34333..34800 |
| *terW* | Tellurium resistance protein TerW | *Serratia*marcescens | 23210..23785 |

Supplementary Figure 1:


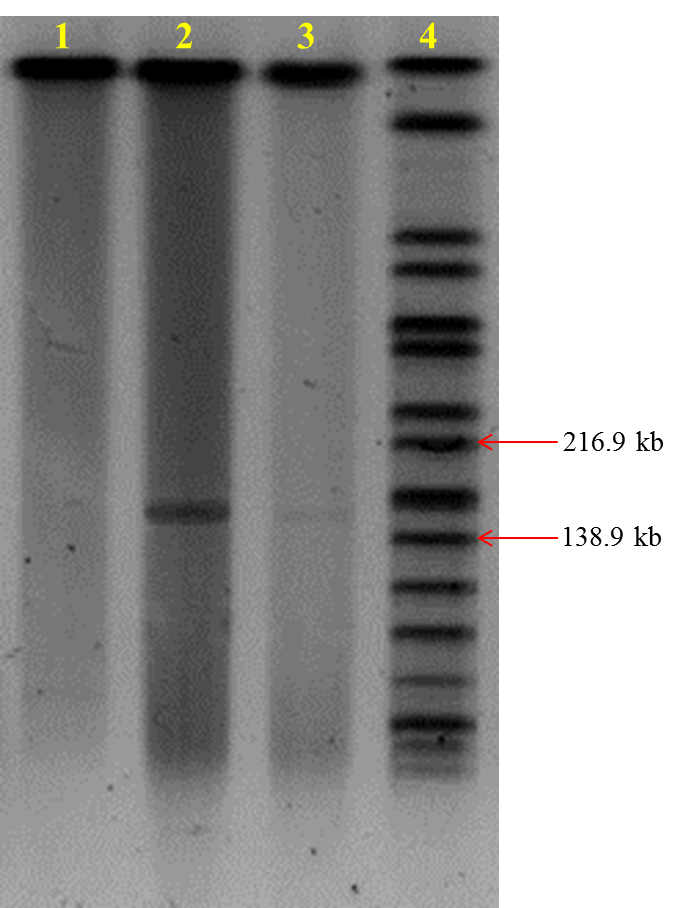


Supplementary Figure 2:


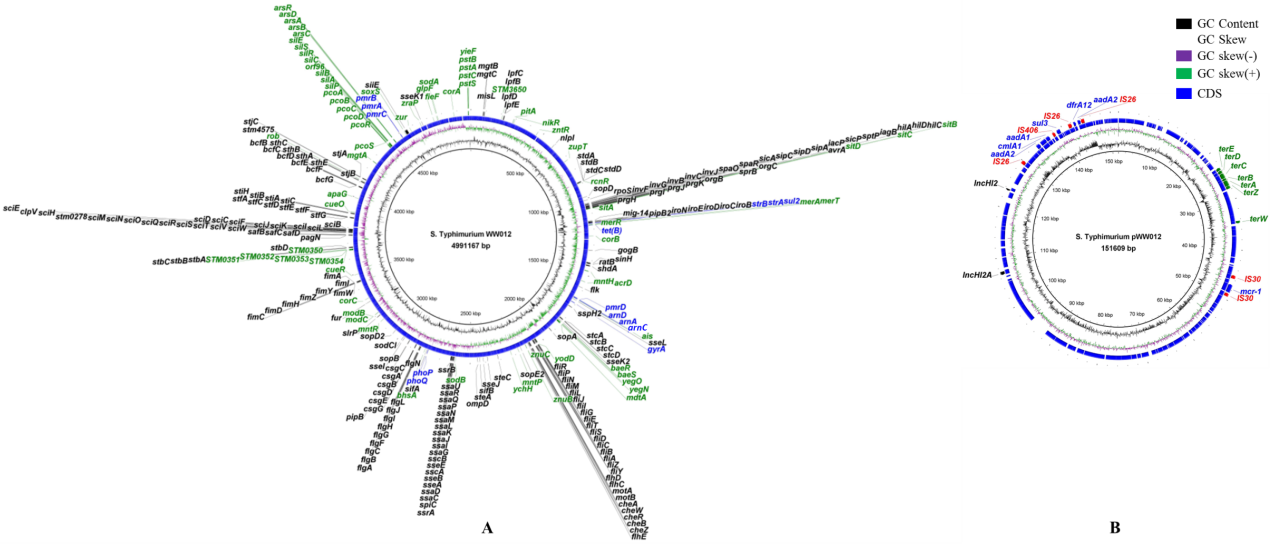


Supplementary Figure 3:


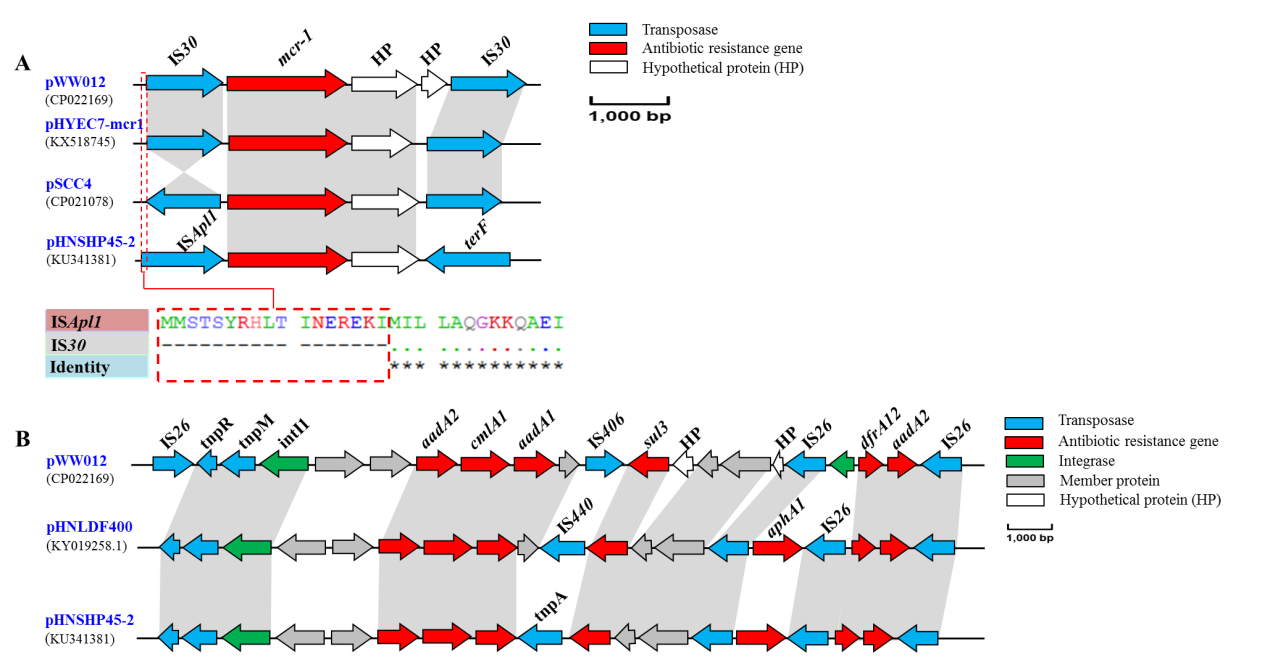

Supplement: FIGURE S1 — S1-PFGE indicating the transfer of single plasmid harboring mcr-1. Lane 1, Escherichia coli J53 (recipient); Lane 2, S. Typhimurium WW012 (donor); Lane 3, CT-E. coli J53(transconjugant); Lane 4, H-9812(PFGE marker strain, S.BraenderupH9812). [file Data_Sheet_1.docx]
